# Evaluation of a Semi-automatic Right Ventricle Segmentation Method on Short-Axis MR Images

**DOI:** 10.1007/s10278-018-0061-3

**Published:** 2018-03-09

**Authors:** Pinar Yilmaz, Karel Wallecan, Wisnumurti Kristanto, Jean-Paul Aben, Adriaan Moelker

**Affiliations:** 1 Department of Radiology and Nuclear Medicine, Erasmus MC University Medical Center, Room Hs-220, P.O. Box 2040, 3000 CA Rotterdam, the Netherlands; 2 Research and Development Department, Pie Medical Imaging, BV, Maastricht, the Netherlands

**Keywords:** Right ventricle, Cardiac MRI, CMR, Segmentation, Quantitative analysis

## Abstract

The purpose of this study was to evaluate a semi-automatic right ventricle segmentation method on short-axis cardiac cine MR images which segment all right ventricle contours in a cardiac phase using one seed contour. Twenty-eight consecutive short-axis, four-chamber, and tricuspid valve view cardiac cine MRI examinations of healthy volunteers were used. Two independent observers performed the manual and automatic segmentations of the right ventricles. Analyses were based on the ventricular volume and ejection fraction of the right heart chamber. Reproducibility of the manual and semi-automatic segmentations was assessed using intra- and inter-observer variability. Validity of the semi-automatic segmentations was analyzed with reference to the manual segmentations. The inter- and intra-observer variability of manual segmentations were between 0.8 and 3.2%. The semi-automatic segmentations were highly correlated with the manual segmentations (*R*^2^ 0.79–0.98), with median difference of 0.9–4.8% and of 3.3% for volume and ejection fraction parameters, respectively. In comparison to the manual segmentation, the semi-automatic segmentation produced contours with median dice metrics of 0.95 and 0.87 and median Hausdorff distance of 5.05 and 7.35 mm for contours at end-diastolic and end-systolic phases, respectively. The inter- and intra-observer variability of the semi-automatic segmentations were lower than observed in the manual segmentations. Both manual and semi-automatic segmentations performed better at the end-diastolic phase than at the end-systolic phase. The investigated semi-automatic segmentation method managed to produce a valid and reproducible alternative to manual right ventricle segmentation.

## Introduction

Assessment of ventricular morphology and function is important in the management of patients with cardiovascular disease. Magnetic resonance imaging (MRI) has been the preferred imaging modality for quantitative analysis of the ventricles [1, 2]. These functional cardiac analyses are usually performed by measuring ventricular volumes at certain cardiac phases, such as at the end-diastolic (ED) and end-systolic (ES) phases, and subsequently calculating the ejection fraction (EF). Ventricular evaluation typically requires the ventricular borders to be segmented first, before further analysis and calculations can be performed. Traditionally in the clinical setting, ventricular border segmentation is performed manually, which is known to be a time-consuming process [3], prone to intra- and inter-observer variability [3–5], and dependent on user experience [3, 5, 6]. Therefore, efforts have been done previously to develop automatic segmentation methods which have been shown to reduce segmentation time [7–9] with comparable or even lower variability than manual segmentations [9, 10].

Automatic segmentation methods have been shown to be beneficial in left ventricle (LV) evaluations on short-axis cine images [8, 11, 12]. Meanwhile, automatic segmentation algorithms for the right ventricle (RV) are less available than for the LV [13]. RV segmentation is more challenging due to its high shape variability and complex movement [14, 15], resulting in a lower performance in terms of variability for both manual and automatic RV segmentations as compared to LV segmentations [3, 16].

Various RV segmentation algorithms have been developed to overcome the inherent difficulties in RV segmentation, ranging from image-driven to model-based algorithms, from semi-automatic algorithms requiring multiple user inputs to fully automatic [14]. While model-based algorithms can be quite powerful, image-driven algorithms are generally regarded to be more robust against pathological and image acquisition variations. Due to the morphologic variations of the RV with regard to its pathological condition [17], robust segmentation algorithms are needed. Semi-automatic algorithms have been shown to outperform fully automatic ones, despite the user interactions needed [14].

In this study, we aim to evaluate a newly developed image-driven semi-automatic RV segmentation method on cardiac short-axis MR images that segment all RV contours in a cardiac phase with minimal user input of one seed contour and with the essential restriction of no manual corrections. Validity and reproducibility of the semi-automatic segmentation will be compared against the manual segmentation.

## Materials and Methods

### Study Population

Twenty-eight consecutive volunteers were included for the current studies. This study was conducted according to the principles of the Declaration of Helsinki (October 2013) and in accordance with the Medical Research Involving Human Subjects Act (WMO). The study received approval by the local institutional review board and each subject gave informed consent.

### MRI

Cardiovascular magnetic resonance (CMR) imaging was performed using a Signa 1.5 T scanner (GE Medical Systems, Milwaukee, WI, USA) with a dedicated 16-channel phased-array cardiac surface coil. A cine volumetric dataset was acquired in short-axis, four-chamber view, and tricuspid valve view directions using a 2D steady-state free precession acquisition sequence with imaging parameters as follows: flip angle 45°, echo time (TE) set at minimal full, repetition time (TR) 3 ms, 8 mm slice thickness, 2 mm interslice gap, number of excitations 0.75, phase field of view percentage 0.65, 12 views/segment, and a matrix of 256 × 256 (resulting in an in-plane resolution between 1.09 to 1.56 mm/pixel). Twenty-four phases per cardiac cycle were reconstructed retrospectively.

### Image Analysis

Both manual and semi-automatic segmentations were performed within CAAS MRV software package (version 4.1; Pie Medical Imaging, BV, Maastricht, the Netherlands) on the short-axis view. The basal slice was inferred from the position of the tricuspid annulus on the four-chamber view [3, 14] and tricuspid valve view. The apical slice was chosen to be the last slice that shows detectable RV activity [14]. The ED and ES phases and the apical and basal slice selections at ED and ES phases were set to be the same for both manual and semi-automatic segmentations. The segmentations were performed at ED and ES phases, on every slice between apical and basal slices.

### Manual Segmentation

Two experienced observers with 7 and 2 years of CMR imaging experience (first and second observer, respectively) independently performed manual segmentations of RV endocardial contours to derive the inter-observer variability. The datasets were anonymized before being presented to the observers. The first observer performed the manual segmentations once (resulting in measurement M1), which serves as reference results. Meanwhile, the second observer performed the manual segmentations twice (resulting in measurements M2a and M2b), in two sessions separated by 2-week period to derive the intra-observer variability (see Table 1 for the overview of measurements). Papillary muscles and trabeculations were treated as part of the blood pool volume.

**Table 1:** Overview of measurements and analyses

Manual measurements	
M1	Manual segmentation by the first observer (reference)
M2a	First attempt of manual segmentation by the second observer
M2b	Second attempt of manual segmentation by the second observer
Semi-automatic measurements	
A1	Semi-automatic segmentation by the first observer
A2a	First attempt of semi-automatic segmentation by the second observer
A2b	Second attempt of semi-automatic segmentation by the second observer
Manual analyses	
Inter-observer variability	M1 vs M2a
Intra-observer variability	M2a vs M2b
Semi-automatic analyses	
Inter-observer variability	A1 vs A2a
Intra-observer variability	A2a vs A2b
Validity	A1 vs M1

### Semi-automatic Segmentation

The semi-automatic RV segmentation algorithm is based on the cellular automata framework which allows every voxel to be labeled as foreground or background based on their signal intensity similarity and their distance to the seeds [18]. This labeling process is implemented using parallel computation techniques and therefore high computation performance can be established. The segmentation algorithm requires prior information of the ED and ES phases, and the apical and basal slices for both the ED and ES phases. At the ED and ES phases, the user is asked to provide a rough RV endocardial contour as a seed in one slice between the identified apical and basal slices. The segmentation is initiated at the slice where the user defined roughly the RV seed contour, which is used by the algorithm during the foreground labeling process. Meanwhile, the background labeling is determined by the algorithm based on features derived from the image itself and cardiac movement extracted from the short-axis slice. After optimizing the seed contour, the resulting RV endocardial contours are propagated towards the apical and the basal slices, taking into account possible misalignment between slices and the RV geometry at a specific cardiac phase (relative to ED and ES phases).

The same two observers performed the semi-automatic RV segmentations, with the same number of segmentations as the manual one (resulting in measurements A1, A2a, and A2b respectively, see Table 1 for the overview of measurements). The observers performed the segmentations independently and were blinded to the results of segmentation until all the data were ready to be processed. Adhering to the common workflow of cardiac examinations, where the LV examinations were performed prior to RV examination, the LV was already segmented before the automatic RV segmentations. The same LV segmentations are provided to all measurements. We have to stress here that for the current study, no manual corrections were performed afterward and the resulting RV contours were used as is.

Ventricular volumes at ED and ES phases were automatically calculated by the software, using the Simpson’s rule:

$$ \mathrm{Volume}=\sum \limits_{i=1}^n{\mathrm{Area}}_i{\mathrm{Thickness}}_i $$

where *i* is the slice level, *n* is the number of slices, Area_*i*_ is the area covered by the RV endocardial contours at the *i*th slice level, and Thickness_*i*_ is the slice thickness at *i*th slice level (including the interslice gap). EF was also automatically calculated using the following equation:

$$ \mathrm{EF}=\frac{{\mathrm{ED}}_{\mathrm{volume}}-{\mathrm{ES}}_{\mathrm{volume}}}{{\mathrm{ED}}_{\mathrm{volume}}}\times 100\% $$

### Statistical Analysis

To assess the performance of the semi-automatic segmentation algorithm, the derived ED and ES ventricular volumes for the right endocardium as well as values for the EF were compared with the manual derived values of the first observer (measurement A1 vs measurement M1). The appropriate term to express the level of agreement between the semi-automatic and manual segmentation is “validity” instead of “accuracy” in view of the fact that no gold standard exists for RV evaluation [19]. The validity was expressed as the mean and standard deviation, 95% limits of agreement (calculated as the mean ± 1.96 * standard deviation), the median, and the interquartile range of the paired differences in each data set. The percentage difference relative to the average value of the manual volumes was also calculated. A preliminary test using the Shapiro-Wilk test on the measurements showed that some of them were not normally distributed. Therefore, a two-tailed Wilcoxon signed-rank test was performed to determine the statistical significance of the observed difference, with *P* < 0.05 considered to indicate significant difference. The Bland-Altman analysis was also performed to visualize the observed differences.

Contours obtained by the semi-automatic segmentation algorithm were also evaluated against the ones of the manual segmentation, using two metrics: the dice metrics (DM) and Hausdorff distance (HD). DM is a measure of area overlap between two contours, using the following equation:

$$ \mathrm{DM}=2\frac{A\cap B}{A+B} $$

where *A* and *B* are the areas enclosed by the two tested contours. The DM ranges from 0 (no overlap) to 1 (perfect overlap). Meanwhile, HD is a measure of maximum distance between two contours expressed in mm, using the following equation:

$$ \mathrm{HD}=\max \left(\underset{x\epsilon X}{\max}\left(\underset{y\epsilon Y}{\min }d\left(x,y\right)\right),\underset{y\epsilon Y}{\max}\left(\underset{x\epsilon X}{\min }d\left(x,y\right)\right)\right) $$

where *X* and *Y* are the two tested contours, *x* and *y* are individual points of *X* and *Y*, respectively, and *d*(*x*,*y*) is Euclidian distance between *x* and *y*.

To put the observed difference of segmentations in perspective, we compared them against the inter- and intra-observer variability as found from the manual segmentations by the two observers. Inter-observer variability of the manual segmentations was obtained by comparing the segmentation results of the first observer and the first result of the second observer (measurement M1 vs M2a) and intra-observer variability by the two segmentation results of the second observer (measurement M2a vs M2b).

To assess the reproducibility of the semi-automatic segmentation algorithm, inter- and intra-observer variability of the semi-automatic segmentations were obtained in a similar way as the manual segmentations, i.e., by comparing automatic segmentation results of the first observer and the first result of the second observer (measurements A1 vs A2a) and by comparing both automatic segmentations results of the second observer (measurements A2a vs A2b), respectively. Validity and reproducibility of the semi-automatic segmentations and reproducibility of the manual segmentations were compared. Table 1 contains the overview of analyses performed in this study.

All statistical analyses were performed using Microsoft Excel 2013 (Microsoft Corp., Redmond, WA). A post hoc statistical power analysis was also performed using the G*Power software [20].

## Results

Table 2 shows the characteristics of the study participants including their ED volumes, ES volumes, and EF measurements. These values are concordant with previous reported RV parameters of healthy subjects [21]. The overall mean age was 30.5 + 6.5 years and 14 volunteers (50%) were males.

**Table 2:** Study characteristics and basic measurements of the right ventricle

	All	Male	Female
*N*	28	14	14
Age (years)	30.5 ± 6.5	30.3 ± 7.6	30.6 ± 5.6
Weight (kg)	72.0 ± 10.6	76.4 ± 9.3	67.5 ± 10.1
Heart rate (bpm)	72.1 ± 12.1	72.1 ± 12.7	72.1 ± 12.0
ED volume (mL)	166.2 ± 37.4	182.8 ± 40.2	149.6 ± 26.5
ES volume (mL)	74.5 ± 21.7	84.6 ± 23.6	64.5 ± 14.2
EF (%)	55.3 ± 6.5	53.8 ± 6.9	56.9 ± 5.8

Values are presented as means ± standard deviation

*N* number of participants, *ED* end-diastolic, *ES* end-systolic, *EF* ejection fraction

**Fig. 1:**
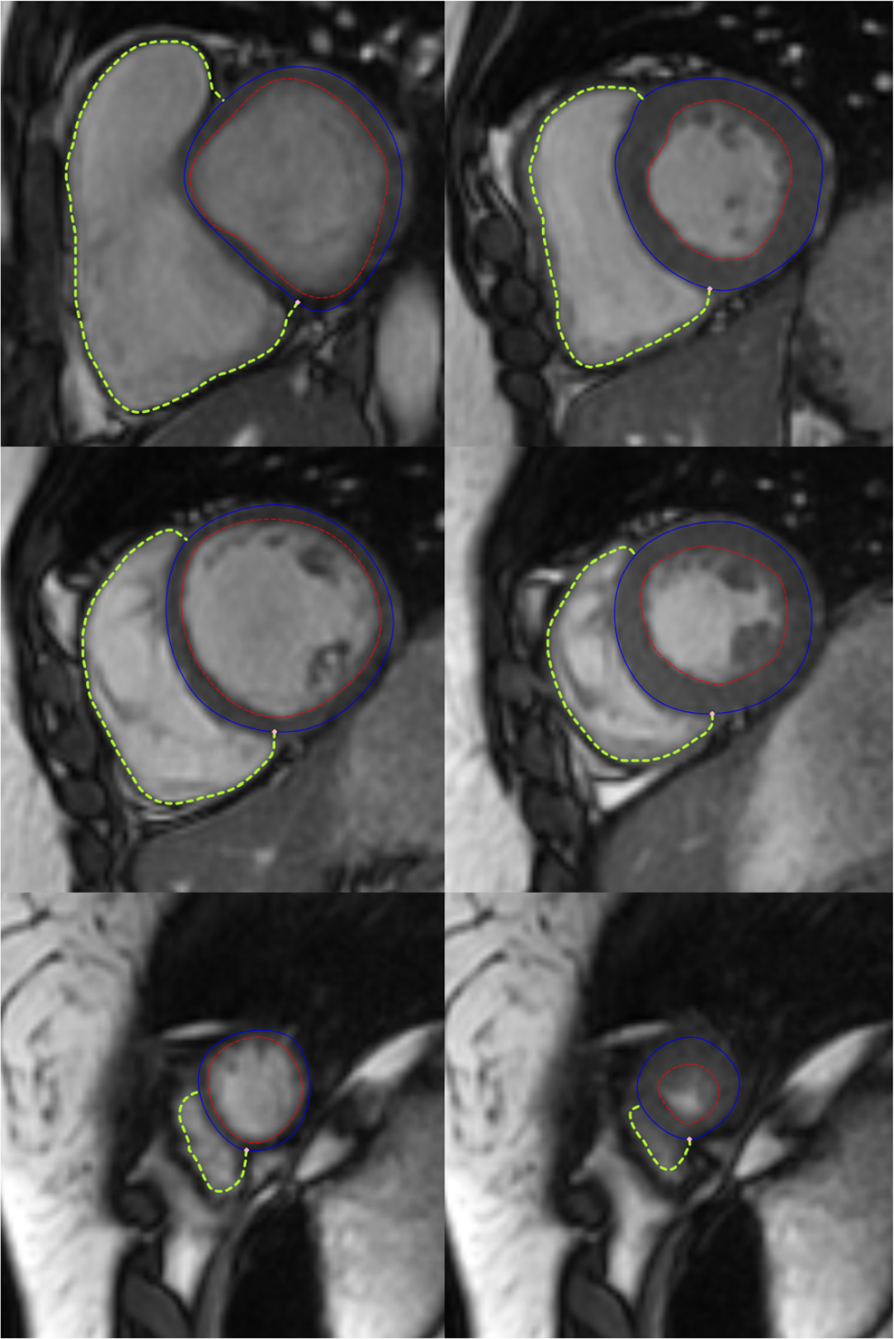
Two-chamber short-axis images of right ventricle automatic segmentation results of a volunteer at end-diastolic (left column) and end-systolic phases (right column) and at basal, mid-cavity, and apical slices (images at top, middle, and bottom rows, respectively). The solid blue and dashed red lines represent the left ventricle epicardial and endocardial contours, respectively. The dashed green lines represent the right ventricle endocardial contours

A typical result of the RV semi-automatic segmentation is shown in Fig. 1. When the LV epicardial contour is available, the RV endocardial contour is shown as attached to the LV epicardial contour, with one of the attach points representing the LV/RV inferior junction point. The average computation time is about 1 s for segmenting RV endocardial contours on all slices within one phase with use of a quad-core 2.8 GHz processor and 16 GB memory on the Windows 7 operating system.

### Manual RV Segmentation Reproducibility

The reproducibility analysis of the manual segmentation is presented in Table 3. All inter- and intra-observer variability results were statistically significant with the exception of the inter-observer EF variability. The inter-observer variability was larger than the intra-observer variability for ED volumes, but smaller for ES volumes and EF.

**Table 3:** Manual right ventricle segmentation reproducibility

	Inter-observer variability (M1 vs M2a)			Intra-observer variability (M2a vs M2b)		
	Value	Percentage	*P*	Value	Percentage	*P*
ED volume	4.62 mL (1.34 to 6.32 mL)	2.74% (0.79 to 3.75%)	0.0000	− 1.33 mL (− 3.33 to 0.16 mL)	− 0.77% (− 1.93 to 0.09%)	0.0036
ES volume	2.23 mL (0.36 to 4.59 mL)	2.95% (0.48 to 6.05%)	0.0108	− 2.48 mL (− 5.04 to 0.25 mL)	− 3.15% (− 6.41 to 0.31%)	0.0012
EF	− 0.72% (− 1.52 to 0.78%)	− 1.30% (− 2.75 to 1.41%)	0.3995	1.01% (− 0.18 to 2.32%)	1.85% (− 0.34 to 4.26%)	0.0148

Value and percentage are presented in median (25th to 75th percentile). Two-tailed Wilcoxon signed-rank test calculated inter- and intra-observer variability with *P* < 0.05 indicating statistical significance

*ED* end-diastolic, *ES* end-systolic, *EF* ejection fraction

### Semi-automatic RV Segmentation Validity

**Fig. 2:**
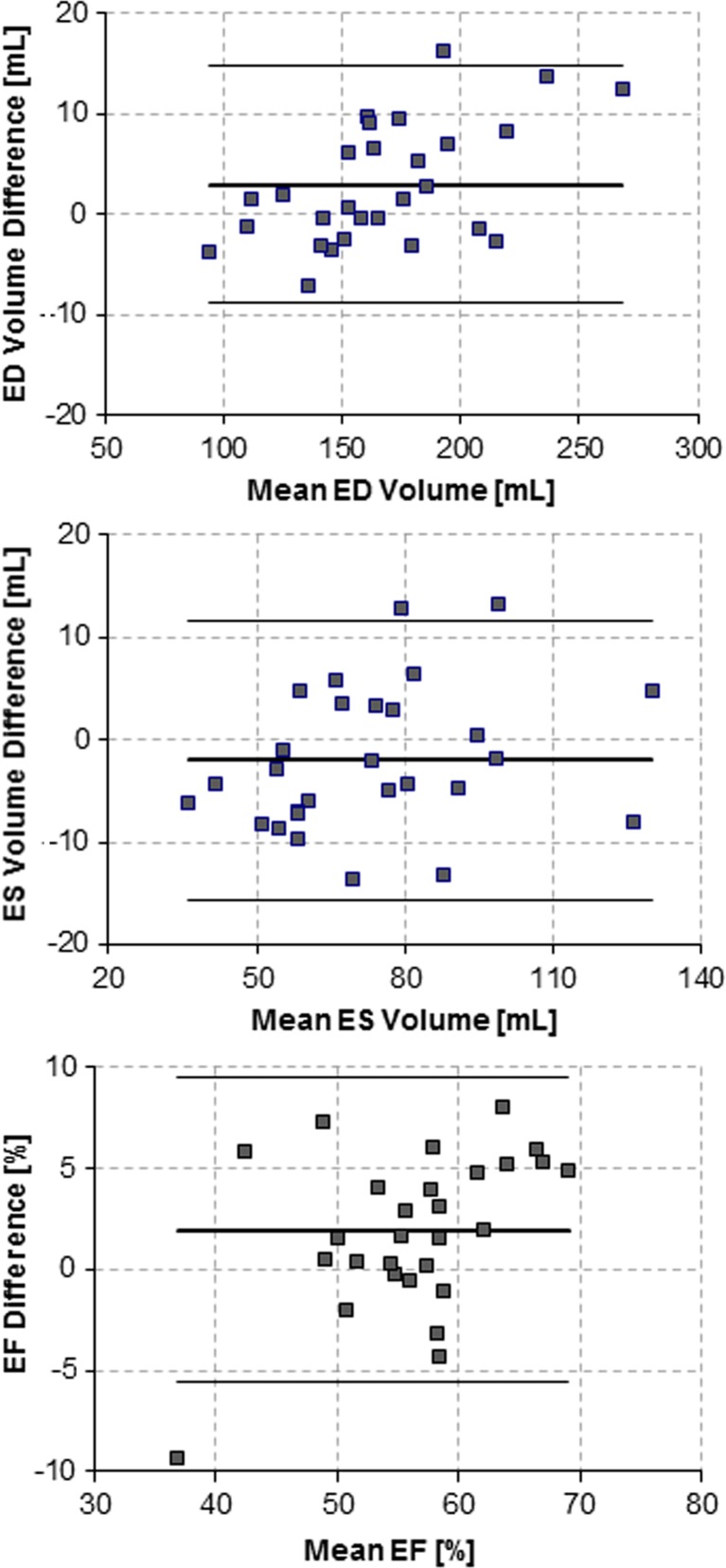
Bland–Altman plots showing the validity analysis of the automatic segmentation method for end-diastolic volume (top), end-systolic volume (middle), and ejection fraction (bottom)

The validity of the semi-automatic segmentations is presented in Tables 4 and 5, and Fig. 2. The semi-automatic segmentation showed good agreement with the manual segmentation (Table 4), with an excellent linear correlation for both ED and ES volumes (*R*^2^ of 0.98 and 0.91, respectively) and slightly less but still good correlation for EF (*R*^2^ of 0.79). The ED volumes had a median difference of less than 2 mL (0.91%), ES volumes showed an underestimation with a median difference less than 4 mL (− 4.84%), and comparison of EF resulted in an overestimation with a median difference of less than 2% (or 3.27% relatively). In comparison to the reproducibility of manual segmentation, the median differences in ED volumes between semi-automatic and manual segmentations were smaller than the manual inter-observer variability. However, in all parameters, the interquartile ranges were larger than the ones of manual intra- and inter-observer variability. Post hoc statistical power analysis on the Wilcoxon signed-rank test yielded 0.62, 0.32, and 0.74 for testing the differences of ED volumes, ES volumes, and EF, respectively. Yet looking in more detail (Table 5), the semi-automatic contours showed good overlap with the manual contours (median DM of 0.95 and 0.87, for ED and ES contours respectively) and revealed small deviations (median HD of 5.05 mm and 7.35 mm for ED and ES contours, respectively).

**Table 4:** Semi-automatic right ventricle segmentation method volumes validity

	Validity analysis of semi-automatic segmentation (A1 vs M1)					Linear regression	
	Value	Percentage			*P*	Equation	*R* ^2^
	Median (25th to 75th percentile)	Mean + SD (limits of agreement)	Median (25th to 75th percentile)	Mean + SD (limits of agreement)			
ED volume	1.51 mL (− 1.66 to 7.25 mL)	2.95 ± 6.02 mL (− 8.85 to 14.75 mL)	0.91% (− 1.00 to 4.36%)	1.77 ± 3.62% (− 5.33 to 8.87%)	0.0153	1.0802 * *x* − 10.391	0.98
ES volume	− 3.61 mL (− 7.04 to 3.34 mL)	− 1.99 ± 6.99 mL (− 15.69 to 11.71 mL)	− 4.84% (− 9.44 to 4.48%)	− 2.67 ± 9.38% (− 21.06 to 15.71%)	0.1434	1.0373 * *x* − 4.7738	0.91
EF	1.81% (0.10 to 5.00%)	1.96 ± 3.86% (− 5.60 to 9.52%)	3.27% (0.18 to 9.03%)	3.55 ± 6.97% (− 10.11 to 17.20%)	0.0120	1.1287 * *x* − 0.0516	0.79

Value and percentage are presented in median (25th to 75th percentile) and in mean ± SD (95% limits of agreement, calculated as mean ± 1.96 * SD). Two-tailed Wilcoxon signed-rank test calculated validity of semi-automatic segmentation with *P* < 0.05 indicating statistical significance

*SD* standard deviation, *ED* end-diastolic, *ES* end-systolic, *EF* ejection fraction

**Table 5:** Semi-automatic right ventricle segmentation method contour validity

	Dice metric		Hausdorff distance	
	Median (25th to 75th percentile)	Mean + SD (limits of agreement)	Median (25th to 75th percentile)	Mean + SD (limits of agreement)
ED	0.95 (0.92 to 0.96)	0.92 ± 0.08 (0.76 to 1.08)	5.05 mm (3.30 to 7.38 mm)	5.82 ± 3.41 mm (− 0.85 to 12.5 mm)
ES	0.87 (0.79 to 0.92)	0.84 ± 0.13 (0.58 to 1.09)	7.35 mm (5.00 to 10.00 mm)	7.76 ± 3.89 mm (0.14 to 15.38 mm)

Dice metric and Hausdorff distance are presented in median (25th to 75th percentile) and in mean ± SD (95% limits of agreement, calculated as mean ± 1.96 * SD). Two-tailed Wilcoxon signed-rank test calculated validity of semi-automatic segmentation with *P* < 0.05 indicating statistical significance

*SD* standard deviation, *ED* end-diastolic, *ES* end-systolic

### Semi-automatic RV Segmentation Reproducibility

For the semi-automatic contour detection, no significant differences could be observed between the first and second observers. The first and second measurements of the second observer showed significant differences in ES volumes and EF. The semi-automatic segmentations showed a highly reproducible segmentation (Table 6), with the largest median difference being lower than 0.5% for all parameters. The inter- and intra-observer variability of the semi-automatic segmentations were noticeably smaller than the manual segmentations.

**Table 6:** Semi-automatic right ventricle segmentation method reproducibility

	Inter-observer variability (A1 vs A2a)			Intra-observer variability (A2a vs A2b)		
	Value	Percentage	*P*	Value	Percentage	*P*
ED volume	0.08 mL (− 0.03 to 0.27 mL)	0.04% (− 0.02 to 0.16%)	0.0795	0.02 mL (− 0.11 to 0.08 mL)	0.01% (− 0.07 to 0.05%)	0.9713
ES volume	0.18 mL (− 0.31 to 1.06 mL)	0.25% (− 0.43 to 1.46%)	0.1059	− 0.36 mL (− 0.89 to − 0.02 mL)	− 0.49% (− 1.21 to − 0.03%)	0.0077
EF	− 0.10% (− 0.74 to 0.25%)	− 0.18% (− 1.29 to 0.43%)	0.1161	0.16% (− 0.02 to 0.58%)	0.29% (− 0.04 to 1.01%)	0.0179

Value and percentage are presented in median (25th to 75th percentile). Two-tailed Wilcoxon signed-rank calculated inter- and intra-observer variability with *P* < 0.05 indicating statistical significance

*ED* end-diastolic, *ES* end-systolic, *EF* ejection fraction

## Discussion

The semi-automatic segmentation results were highly correlated with the manual segmentation results with lower inter- and intra-observer variability than observed in the manual segmentations. The reproducibility of the manual segmentations was in line with previously reported values (Table 7). In comparison to the reproducibility of these previous studies, the validity level of the semi-automatic segmentations was generally on par or better and the inter- and intra-observer variability were considerably lower. Out of all the values listed in Table 7, the results presented in the current study are best comparable to the results of Caudron et al. [3], since there was consensus within and between the observer(s) on basal and apical slices and ES phase.

**Table 7:** Publications on the reproducibility of right ventricle segmentation

Publication	Inter-observer variability			Intra-observer variability			Method^c^	Subjects^d^	Trab. & Pap.^e^
	EDV^a^ mL (%)	ESV^a^ mL (%)	EF^b^ %	EDV^a^ mL (%)	ESV^a^ mL (%)	EF^b^ %			
Boxt [22]	(6.1)	(3.6)	n.a.	(5.1)	(3.7)	n.a.	M	H	n.a.
	(5.8)	(11.4)	n.a.	(10.3)	(9.8)	n.a.	M	P	n.a.
Alfakih [1]	− 5.8 ± 8.1	n.a.	2.9 ± 5.8	− 1.3 ± 5.8	n.a.	1.5 ± 3.0	M	H	n.a.
Beygui [23]	− 1.6 ± 7.3	0.1 ± 5.5	− 1.5 ± 4.3	− 1.1 ± 8.5	− 1.2 ± 6.0	0.5 ± 5.2	M*	Mix	E
Hudsmith [16]	n.a.	n.a.	− 2.8 ± 6.2	n.a.	n.a.	0.1 ± 3.2	M	H	E
Mooij [5]	12.7 ± 11.8	8.4 ± 12.0	− 1.2 ± 4.4	n.a.	n.a.	n.a.	M	Mix	n.a.
Winter [24]	− 7.0 ± 21.0	− 5.0 ± 18.0	− 0.1 ± 2.7	− 5.0 ± 13.0	− 4.0 ± 9.0	0.1 ± 2.0	M	P	I
	− 8.0 ± 24.0	− 4.0 ± 20.0	− 0.6 ± 5.3	− 4.0 ± 27.0	− 0.02 ± 8.0	0.02 ± 3.2	M	P	E
Luijnenburg [4]	4.0 ± 7.1	6.8 ± 6.4	− 2.7 ± 3.0	0.9 ± 5.3	3.4 ± 3.4	− 1.6 ± 1.9	M	P	E
Caudron [3]^f^	n.a.	n.a.	2.4 ± 3.0	n.a.	n.a.	− 0.7 ± 2.9	M	P	I
Sardanelli [25]	n.a.	n.a.	2.3 ± 14.9	n.a.	n.a.	− 3.9 ± 12.5	M	P	E
	n.a.	n.a.	0.7 ± 11.7	n.a.	n.a.	3.7 ± 13.3	SA+	P	E
Lorenz [26]	− 0.0 ± 4.2	3.0 ± 6.5	n.a.	0.2 ± 5.6	− 0.7 ± 2.6	n.a.	SA+	H	E
Catalano [27]	5.0 ± 17.0	2.0 ± 12.0	2.0 ± 9.0	− 5.0 ± 16.0	− 2.0 ± 10.0	− 1.0 ± 10.0	SA+	Mix	I

*EDV* end-diastolic volume, *ESV* end-systolic volume, *EF* ejection fraction, *Trab*. *& Pap*. trabeculations and papillary muscles, *n*.*a*. data were not available or not presented for comparison with current study, *M* manual segmentation, *H* healthy subjects, *P* patients, M* manual segmentation with pre-segmented left ventricle, *Mix* mixed between healthy subjects and patients, *E* excluded in ventricle volume, *I* included in ventricle volume, *SA+* semi-automatic segmentation with manual correction afterwards

^a^Data displayed without brackets are value differences presented in mean ± standard deviation in mL; while data displayed in the brackets are percentage differences

^b^Data displayed are value differences in percentages presented in mean ± standard deviation

^c^Segmentation method used by the observer to delineate the right ventricle

^d^Subjects in each study

^e^Inclusion or exclusion of trabeculations and/or papillary muscles in the calculation of right ventricle volume

^f^Caudron [3]: inter- and intra-observer variability when the observers have chosen the same basal and apical slices and end-systolic phase

Difficulties in RV segmentation at ES phases are well-known [3, 14] and attributed to partial volume effects [14] and to the more complex anatomical RV structure [24], especially at ES phase with maximum contraction of the right ventricle resulting in more compacted trabeculations and papillary muscles, limiting the segmentation process. Despite the slight underestimation of the volume measurements at ES phases in our semi-automatic segmentation method, the validity of EF still fell on average within 2% range with 95% limits of agreement smaller than ± 10% (in values difference). In a recent RV segmentation challenge, held at the Medical Image Computing and Computer Assisted Interventions (MICCAI) 2012 conference [14] and joined by seven imaging groups, the best performing algorithm managed to produce EF measurement with validity in the range of 6% with the general results producing 95% limits of agreement around ± 20% (in values difference). Several newly developed automatic RV segmentation algorithms using the same dataset as the RV segmentation challenge have been published [28–31]. An improvement of the EF validity results has been reported to be around 2% with the 95% limits of agreement slightly higher than ± 10% [28].

Manual segmentations have been known to be time-consuming, taking from 5 min [25] up to 54 min per patient [5] depending on factors such as user experience and contouring methods. (Semi-)automatic segmentation methods that assist an analyst during this process within a small amount of time and yielding valid and reproducible results will be very beneficial for the physician in the clinical workflow. Our segmentation method allows valid and reproducible results, with one roughly user-drawn seed contour and within approximately 1 second of computation time to perform segmentation at one cardiac phase. These virtues are preferred in clinical settings for robustness and reduction in examination time [14]. We would like to argue that the functionality provided by the currently evaluated semi-automatic segmentation method provides an optimal balance between the ease-of-use and the algorithm performance.

Evaluating LV and RV concurrently is beneficial because problems with either side of the ventricles frequently involves the other [32]. The common approach for concurrent LV and RV evaluation is by simultaneously showing both LV epicardial and RV endocardial contours at the septum site [1, 33] which might introduce an error when there is an overlap or a gap between the two contours, i.e., the left ventricular epicardial border may extend into the RV or vice versa. In the currently investigated software package, the RV endocardial contour is attached to the LV epicardial contour, such that the RV endocardial and the LV epicardial contours effectively share the septum and avoid the aforementioned problem. Another advantage of this approach is that the structures attached to the septum, such as the septomarginal trabecula [34], will be automatically included into the right endocardial area. A previous study has shared this way of reasoning and presented a similar approach [23]. It is debatable whether to include or to exclude the trabeculations and papillary muscles into RV cavity delineations. However, inclusion of these structures is recommended to promote reproducibility [24].

The semi-automatic RV segmentation method is evaluated on short-axis cine MRI images. The use of short-axis image orientation for RV analysis promotes efficiency because with one image set both ventricles can be analyzed [35]. However, since short-axis cine MRI is designed for LV analysis, it may not be fully optimized for RV analysis. One of the drawbacks is that the tricuspid valve may not be present in the imaging plane, making it difficult to distinguish ventricles from atria at the basal slices [35–37] and to localize the RV outflow tract which may be out of plane [38]. Difficulties in segmenting these two structures at the basal region have been reported as one of the contributing factors in lower reproducibility in RV segmentation [25]. The Society for Cardiovascular Magnetic Resonance recommends the use of transaxial cine MRI images for RV volumetric analysis [39]. Despite the difficulties in distinguishing blood and myocardium border at inferior RV wall, RV segmentation on transaxial cine MRI images has shown to provide higher reproducibility, probably due to the easiness of locating pulmonary and tricuspid valves [35]. However, such improvement might be too small to be clinically significant and warrant an extra RV examination on transaxial images in addition to the normal CMR examination on short-axis images [40]. Several alternative imaging orientations have been suggested to improve RV segmentation reproducibility, such as: a modified RV short-axis view which is oriented to the RV outflow [36], due to the same advantage of easiness in locating the tricuspid valve; or an acquisition of six slices rotated along the long-axis of the RV, each forming 30° wedge to each other [38]. Variation of short-axis plane orientations exists, and a short-axis orientation perpendicular to the septum has been recommended to obtain optimal RV and LV measurements [41]. One way to mitigate the problem of choosing the most basal slice in short-axis images is by using other image orientation which is perpendicular to them, such as the four-chamber view [3, 14]. Accordingly, this study used the four-chamber view but also the tricuspid valve view, which clearly depicted the tricuspid annulus, to locate the basal slice in the short-axis view. One of the evaluation parameters set up in this study is the reproducibility of the semi-automatic segmentation method. By pre-selecting the basal slices and setting the pre-selected level of basal slices similar for all measurements of the same dataset, thus removing one source of variability [3], the reproducibility analysis was able to be focused on the performance of the method.

There are several limitations in our study. First, the data used for evaluation were acquired on healthy volunteers and using one specific set of image acquisition protocols and MRI scanner. Various cardiovascular diseases can affect RV morphology and structures [24, 42] which may hamper the performance of automatic segmentation methods. However, the employed algorithm relies on features present in the image data itself and it has been pointed out [14] that such a method should be invariant to pathological cases and image acquisitions. Nevertheless, future validation study is still needed to evaluate the performance of this segmentation method on variations of datasets.

## Conclusions

In conclusion, the investigated semi-automatic RV segmentation method managed to produce a valid and reproducible alternative to manual RV segmentation, with limited number of user interactions and computation time.
